# A right frontal network for analogical and deductive reasoning

**DOI:** 10.1093/brain/awaf062

**Published:** 2025-04-16

**Authors:** Joseph Mole, James K Ruffle, Amy Nelson, Edgar Chan, Tim Shallice, Parashkev Nachev, Lisa Cipolotti

**Affiliations:** Department of Neuropsychology, National Hospital for Neurology and Neurosurgery, London WC1N 3BG, UK; Institute of Neurology, University College London, London WC1N 3BG, UK; Institute of Neurology, University College London, London WC1N 3BG, UK; Lysholm Department of Neuroradiology, National Hospital for Neurology and Neurosurgery, London WC1N 3BG, UK; Institute of Neurology, University College London, London WC1N 3BG, UK; Department of Neuropsychology, National Hospital for Neurology and Neurosurgery, London WC1N 3BG, UK; Institute of Neurology, University College London, London WC1N 3BG, UK; Institute of Cognitive Neuroscience, University College London, London WC1N 3AZ, UK; Cognitive Neuropsychology and Neuroimaging Lab, International School for Advanced Studies (SISSA-ISAS), 34136 Trieste, Italy; Institute of Neurology, University College London, London WC1N 3BG, UK; Department of Neuropsychology, National Hospital for Neurology and Neurosurgery, London WC1N 3BG, UK; Institute of Neurology, University College London, London WC1N 3BG, UK

**Keywords:** reasoning, intelligence, frontal lobes, executive functions, network lesion-symptom mapping

## Abstract

Two of the most well-studied types of reasoning are analogical reasoning (AR) and deductive reasoning (DR). Yet, our understanding of the relationship between reasoning abilities and their neuroanatomical basis remains surprisingly limited. We aimed to conduct fine-grained anatomical mapping of performance on tests of AR, DR and fluid intelligence (Gf), in a large sample of patients with unilateral focal frontal or posterior lesions and healthy controls.

We assessed 247 prospectively recruited patients using two new tests: the Analogical Reasoning Test (ART) and the Deductive Reasoning Test (DRT); and the best-established measure of Gf: Raven’s Advanced Progressive Matrices (RAPM). Non-parametric Bayesian stochastic block modelling was used to reveal the community structure of lesion deficit networks, disentangling functional from confounding pathological distributed effects.

ART and DRT performance was significantly impaired in patients with frontal lesions [ART: *F*(2,238) = 18.93; *P* < 0.001; Frontal group worse than Posterior group and healthy controls, both *P* < 0.001; DRT: *F*(2,387) = 18.491; *P* < 0.001; Frontal group worse than healthy controls, *P* < 0.01]. Right frontal effects were evident on both tests. Thus, on the ART, right frontal patients were more impaired than left (*P* < 0.05). On the DRT, right frontal patients were more impaired than left frontal patients on questions with indeterminate solutions (*P* < 0.05) but not on questions with determinate ones. Non-parametric Bayesian stochastic block modelling implicated a right frontal network in ART and DRT performance. Strikingly, we found that this network was also implicated in performance on RAPM.

Our study represents the most robust investigation of AR and DR in the focally injured brain. Our findings imply that a right frontal network is critical. The ART and DRT appear to be promising new clinical tests, capable of evaluating reasoning abilities and identifying right frontal lobe dysfunction.

## Teaser

Mole *et al.* have investigated the neural basis of analogical and deductive reasoning abilities by administering two new tests of reasoning to 247 patients with focal brain damage. A network of right frontal brain areas was found to be critically important to performance on both tests.

**See Reverberi *et al.* (https://doi.org/10.1093/brain/awaf117) for a scientific commentary on this article.**

## Introduction

Reasoning skills are central to many of humanity’s greatest intellectual endeavours: mathematics, philosophy and science, to name but a few.^1^ Unsurprisingly, reasoning has itself been the subject of intense academic interest since the dawn of scientific enquiry.^2^

Two of the most well-studied types of reasoning are analogical reasoning (AR) and deductive reasoning (DR). Analogical reasoning is the process of identifying similarities between relationships (e.g. ‘1,2,3’ is most similar to ‘5,6,7’ or ‘6,5,7’?).^3^ This form of reasoning is held to underpin our ability to solve problems by transferring information from one set of relationships to another. In contrast, DR is the ability to derive a logical conclusion from a set of premises that are held to be true. This form of reasoning is thought to be critical for solving problems with determinate solutions (e.g. ‘If Mary is braver than John and John is braver than Michael is Mary braver than Michael?’), as well as identifying when solutions cannot be determined (e.g. ‘If Sarah is smarter than Diane and Sarah is smarter than Heather is Diane smarter than Heather?’).^4^

Our understanding of the relationship between reasoning abilities and other cognitive processes, and their neuroanatomical basis, remains surprisingly limited.^5^ Several influential theories have proposed close links between reasoning abilities and fluid intelligence (Gf). Gf is the ability to solve challenging novel problems when prior learning or accumulated experience are of limited use.^6^ The influential Cattell–Horn–Carroll theory proposes that reasoning skills are the hallmark of Gf.^6^ Within this theory, Gf is considered to be a ‘broad’ cognitive domain comprising several qualitatively different reasoning abilities, such as: inductive reasoning—encompassing AR, sequential reasoning—encompassing DR and quantitative reasoning—encompassing the ability to reason using mathematical concepts.

Duncan *et al*. proposed that reasoning abilities and Gf are underpinned by a network of mainly bilateral frontal and parietal areas, termed the ‘multiple-demand network’ (MD).^7^ This proposal was largely based on functional magnetic resonance imaging (fMRI) studies in healthy adults reporting MD activation during performance on tests of reasoning, Gf and executive functioning.^7^ According to this proposal, the function of the MD is best conceptualized as a single cognitive process, namely Gf. Similarly, the parieto-frontal integration theory (P-FIT) proposed that a rather large network of bilateral parieto-frontal brain areas, as well as temporal and occipital cortex, underpins performance on tests of reasoning, Gf, general and crystallized intelligence.^8^

Reasoning skills and Gf have also been thought as key mental processes involved in ‘active thinking’, the cognitive processes necessary for confronting situations where we do not respond routinely but, rather, effectively address novel problems.^9^ Active thinking also encompasses a variety of other executive abilities, such as those involved in abstraction, switching lines of thought and formulating strategies. The general theoretical position is that different types of active thinking processes are supported by a set of potentially separable cognitive processes, underpinned by separate lateralized frontal lobe networks. In line with this, we recently reported that a rather localized right frontal network underpins performance on one of the most well-established measures of Gf [Raven’s Advanced Progressive Matrices (RAPM)].^10^ We hypothesized that this network may be critical to the ability to make high-level inferences based on the perception of the progression of a pattern. In contrast, we have reported preliminary findings suggesting that a left frontal network may support ‘task setting’: the ability to formulate a mental programme to guide action, which is needed, among other processes, when solving multi-stage calculations.^11^

Most existing studies that have investigated the anatomical substrates of AR and DR have used fMRI in healthy adults and reported rather mixed results. Several fMRI studies of AR have reported activation in the left rostrolateral prefrontal cortex^12,13^ However, others have reported bilateral, or even right, frontal activation.^14-19^ The results from neuroimaging investigations of DR have implicated various frontal and posterior areas, in both hemispheres.^20-23^ However, it remains unclear whether these conflicting results can be accounted for by methodological inconsistencies and/or the inherently correlative nature of fMRI evidence or, instead, may hint at the existence of specialized networks supporting different aspects of reasoning.

Some authors have suggested that brain areas in both hemispheres may be critical for different types of reasoning. For example, Goel *et al*.^4^ proposed that the left frontal lobe supports reasoning in situations where a solution can be determined. Instead, the right frontal lobe supports reasoning in situations where a solution is indeterminate. Prado *et al*.^24^ argued that the left inferior frontal gyrus and basal ganglia underpin performance on categorical DR questions (e.g. ‘All As are Bs. All Bs are Cs. Therefore, all As are Cs’), the left precentral gyrus support performance on propositional DR questions (‘If there is an A, then there is a B. There is an A. Therefore, there is a B’), whereas the right middle frontal gyrus and posterior parietal cortex support performance on relational DR questions (e.g. ‘A is to the left of B. B is to the left of C. Therefore, A is to the left of C’). Other authors have placed greater emphasis on the left hemisphere. For example, Holyoak and Monti^25^ suggested that reasoning abilities are reliant upon several different cognitive processes involved in the representation and integration of relationships, and each process is supported by different brain areas localized in a predominantly left-lateralized frontoparietal network. Wang *et al*.^26^ suggested that DR may be supported by a distributed network, encompassing left frontal/parietal cortices and subcortical structures, and a ‘core’ network, comprising the left inferior frontal gyrus, insula and cingulate. Reverberi *et al*.^27^ argued that left ventrolateral frontal, left inferior lateral frontal and superior medial frontal cortices are critical for syllogistic deductions, but that the pattern of activation depends upon the behavioural strategies employed. Thus, while several proposals may account for the apparently conflicting results from neuroimaging studies, it remains unclear which brain areas are critical.

An important caveat of fMRI techniques is that they cannot reliably differentiate regions activated in the exercise of a cognitive ability from those necessary for it.^28^ For example, an observation of activation in a given brain area during performance on a cognitive test—even if reliably replicated over multiple studies—does not necessarily imply that this area has a direct bearing on performance.^29^

To overcome the limitations of neuroimaging methods, several authors have investigated reasoning abilities using transcranial magnetic stimulation (TMS). For example, Boroojerdi *et al*.^30^ reported that repetitive TMS (rTMS) over the left frontal lobe was associated with faster response times on a test of AR, without reducing accuracy. Tsujii *et al*.^31^ reported that bilateral superior parietal lobule stimulation disrupted performance on abstract and incongruent categorical DR questions, left inferior frontal gyrus stimulation disrupted performance on congruent categorical DR questions and right inferior frontal gyrus stimulation disrupted performance on incongruent DR questions. However, methodological issues complicate the interpretation of these findings. For example, it is now understood that TMS has limited coverage and its effects are not necessarily focal.^32^ Furthermore, it is technically difficult to disrupt spatially extended locations simultaneously with TMS, rendering networked neural substrates hard to delineate with confidence.

Lesion studies on patients with focal, unilateral, brain damage, caused by pathologies such as brain tumour or stroke, offer a unique opportunity to further our understanding of the neurocognitive architecture underpinning reasoning.^9^ In contrast to studies using functional neuroimaging, focal lesion studies provide causal—rather than correlational—evidence.^33^ In comparison with studies using TMS, focal lesion studies offer full brain coverage and an opportunity to investigate spatially extended effects. Yet, such studies are surprisingly rare. Moreover, existing studies have investigated very small patient samples, hence they are likely to be biased and have random variation. Indeed, they have reported different findings. Thus, to the best of our knowledge, only three lesion studies have investigated AR. Langdon and Warrington^34^ found no significant difference on verbal and spatial AR tests between patients with unilateral anterior (left *n* = 14; right *n* = 12) or posterior (left *n* = 24; right *n* = 26) lesions. Notably, the anterior lesions extended into posterior areas, limiting interpretation of results. Schmidt *et al*.^35^ investigated performance on a verbal AR task in 34 stroke patients with unilateral left (*n* = 17) or right (*n* = 17) hemisphere lesions. Only 16 patients’ lesions involved the frontal lobes. Voxel-based lesion-symptom mapping (VLSM) revealed that several frontal and temporal areas were critical for performance. Urbanski *et al*.^36^ reported that 27 patients with frontal lesions (left *n* = 14; right *n* = 9; bilateral *n* = 4) were less accurate than healthy controls (HCs) on a test of AR. VLSM and tractography analyses revealed that AR was impaired following damage to the left rostrolateral prefrontal cortex and some of its long-range connections. Crucially, however, in the studies of both Schmidt *et al*.^35^ and Urbanski *et al*.,^36^ several frontal areas were not lesioned in a sufficient number of patients to be analysed using VLSM. This, and the now well-established flaws of mass univariate lesion-deficit mapping, especially with data of this minimal scale, prevent strong inferences from being drawn about the neuroanatomical basis of AR.^37,38^

To the best of our knowledge, only two focal lesion studies have investigated DR. Reverberi *et al*.^39^ reported that patients with left lateral (*n* = 10) and superior medial frontal (*n* = 18), but not right frontal (*n* = 8), lesions were significantly less accurate than HCs at solving DR questions. Notably, all DR questions included in this study had determinate solutions. Goel *et al*.^4^ reported that left frontal patients (*n* = 9) were significantly less accurate at solving relational DR questions with determinate solutions, when compared with right frontal patients (*n* = 9) and HCs (*n* = 22). In contrast, right frontal patients were significantly less accurate at solving DR questions with indeterminate solutions, when compared with left frontal patients and HCs. The authors argued that these results support the proposal that the left and right frontal lobes underpin determinate and indeterminate DR, respectively. However, a significant limitation of this study is that over two-thirds of the patients suffered from penetrating head injuries, which frequently lead to diffuse axonal injury, typically undetected by conventional neuroimaging measures.^40^ Hence, these lateralized effects must be treated with caution.

Here, we conduct fine-grained anatomical mapping of performance on tests of both AR and DR, in a large sample of 247 patients with unilateral focal frontal or posterior lesions. We designed two new computerized tests: the AR Test (ART) and DR Test (DRT). Given the putatively distributed nature of the target neural substrate, we employed graph lesion-deficit mapping, founded on non-parametric Bayesian stochastic block modelling, allowing us to infer its network organization.^10^ This approach has the power to disentangle distributed patterns of neural dependence from complex anatomical patterns of pathological damage itself, providing a rigorous test of network-framed hypotheses.

## Materials and methods

### Participants

Two-hundred and forty-seven patients with unilateral, focal lesions were prospectively recruited from the National Hospital for Neurology and Neurosurgery (NHNN; see Supplementary material for inclusion criteria). Of these 247 patients, 176 had lesions that fell within frontal (*n* = 102; left frontal 47; right frontal 55) or posterior (*n* = 74; left posterior 32; right posterior 42) areas (i.e. ≥70% of the total lesion, calculated following segmentation of MRI or CT scans (see Supplementary material).^41^ There was no significant difference between tumour and stroke patients for mean time between injury and neuropsychological assessment (*P* = 0.30). Handedness was recorded in 239 of the 247 patients; of these, 232 (97.1%) were right-handed and 7 (2.9%) were left-handed.

Eighty-one HCs, with no neurological or psychiatric history, were recruited to match patients as closely as possible for age, gender, years of education and National Adult Reading Test (NART) scores.^42^ The study was approved by The NHNN and Institute of Neurology Joint Research Ethics Committee and conducted in accordance with the Declaration of Helsinki.

### Behavioural investigations

Participants were assessed with tests administered and scored in the published standard manner.

#### Background tests

Premorbid optimal level of functioning was assessed using the NART, perception using Incomplete Letters, naming using the Graded Naming Test (GNT),^43^ receptive language using the last 12 items from the Test for Reception of Grammar (TROG)^44^ and Gf assessed using RAPM.^45^ Two widely used executive tests, known to require processes distinct from Gf were administered:^46,47^ the Phonemic fluency test (total number of ‘S’ words generated, excluding errors)^46^ and the Stroop test (total number of ink colours correctly named in 2 min, or pro-rated if completed within 2 min).^47^

#### Novel behavioural tasks

The ART and DRT ran on 10.5-inch iPad Pro tablet computers, using script-based psychometric software (https://www.neurobs.com/).

#### Analogical reasoning task

**Figure 1:**
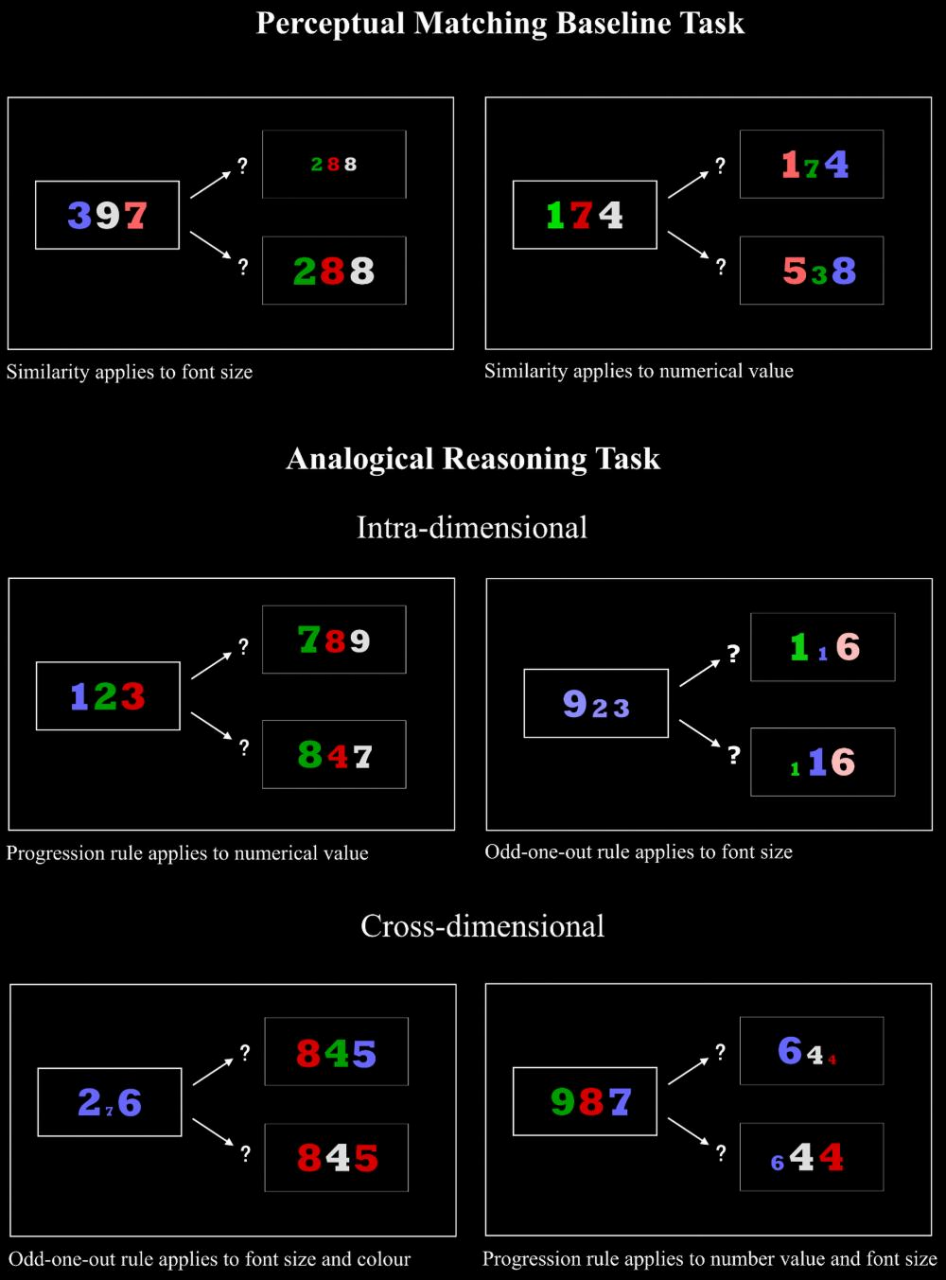
**Example of questions from the analogical reasoning task**.

Participants were administered the ART and a Perceptual Matching Baseline Task (see Fig. 1). Both used the same type of stimuli and design, and were inspired by tasks developed by Urbanski *et al*.^36^ (see Supplementary material for a detailed description of the tasks).

The Perceptual Matching Baseline Task began with three instruction questions, followed by 12 test questions. For each question, three sets of coloured numbers appeared on the left (the source set) and two sets appeared on the right (the target sets). Participants were told to touch the set of numbers on the right that looked most similar to the set on the left. Participants were then administered 12 test questions and instructed to respond quickly and accurately.

The ART was administered immediately afterwards. This began with six instruction questions, demonstrating the correct answers. Participants were told to again indicate which of the sets of numbers on the right is most alike the set on the left. However, this time they should do this based on whether they follow the same abstract rule.

Analogies were based on two rules: ‘Progression’ and ‘Odd-one-out’ (OOU). On Progression questions, the source and correct target set were similar because there was an increase or decrease in numerical value, intensity of colour or size. On OOU questions, the source and correct target set were similar because one of the numbers was the OOU in terms of numerical value, colour or size.

Half of the questions were ‘intra-dimensional’—the analogy rule applies to the same stimuli feature (e.g. colour) in the source and target set—and half were ‘cross-dimensional’—the rule applies to different stimuli features (e.g. colour and size).^36^

Following the instruction questions, participants were administered 24 test questions. They were instructed to respond quickly and accurately and feedback was provided.

We analysed the percentage of correct responses (i.e. the number of correct responses/total number of questions × 100) on the Perceptual Matching Baseline Task and ART. Percentage of correct responses was also calculated separately for Progression (*n* = 12), OOU (*n* = 12), intra-dimensional (*n* = 12) and cross-dimensional questions (*n* = 12).

#### Deductive reasoning task

The DRT comprised a series of 24 relational deductive reasoning questions (see Supplementary material for a detailed description). Participants were told that their task was to determine if the third sentence follows logically from the first two sentences, by pressing either ‘Yes’ or ‘No’.

Participants were presented with two practice questions. The first was determinate and logically valid (e.g. IF Lucy is braver than Samantha, AND Samantha is braver than Rebecca, THEN Lucy is braver than Rebecca?). The second was indeterminate and logically invalid (e.g. IF Chris gets better grades than Helen: AND Chris gets better grades than Dorothy; THEN Helen gets better grades than Dorothy?). Feedback was presented after both questions.

Participants were then administered 24 test questions, with no feedback. Participants were instructed to indicate, quickly and accurately, whether each question was logically valid. There was an equal number of valid and invalid questions. All valid questions were necessarily determinate. Half of the invalid questions were determinate and half were indeterminate.

We analysed the overall percentage of correct responses (i.e. the number of correct responses/total number of questions × 100). The percentage of correct responses was also calculated separately for determinate (*n* = 18) and indeterminate questions (*n* = 6).

### Neuroimaging investigations

Imaging data were available for 237 patients (*n* = 232 MRI, *n* = 5 CT; *n* = 95 Frontal, *n* = 71 Posterior). The procedure for lesion tracing and classification is described elsewhere, and summarized in the Supplementary material. The lesion distribution is displayed in Supplementary Fig. 2.

### Behavioural analysis

Behavioural analyses were conducted on the 176 patients with lesions falling within frontal or posterior areas and HCs using SPSS version 29 (https://www.ibm.com/spss). Neuropsychological data were assessed for skewness and kurtosis and tested for normality using the Shapiro–Wilk test.

Frontal, Posterior and HC groups were compared on demographic variables, using ANOVA and Fisher’s exact test, and performance on neuropsychological tests, ART and DRT, using analysis of covariance (ANCOVA), controlling for age. Following significant differences, *post hoc* tests with Bonferroni correction (alpha 0.05/3 = 0.016) compared Frontal versus Posterior, Frontal versus HC and Posterior versus HC groups.

To investigate potential lateralized frontal effects, Left frontal and Right frontal groups were compared on demographic variables, using *t*-tests and Fisher’s exact tests, and performance on neuropsychological tests, ART and DRT, using ANCOVA, controlling for age. Finally, as it has been reported previously that AR performance is more accurate on intra-dimensional than cross-dimensional questions,^36^ pairwise comparisons were made using ANCOVA, to compare performance on intra- versus cross-dimensional questions, while controlling for age, in the sample as a whole and in Frontal, Posterior and HC groups separately.

### Neuroimaging analysis

Capturing anatomically distributed neural dependence—and disentangling it from the pathology-driven patterns of damage that reveal it—requires a model of the interactions between anatomical loci. Here, we employed a principled and previously validated approach based on statistical models of graphs.^10,48^ In brief, the brain is modelled as a network, where each node is an anatomical location and each edge indexes the extent to which its connected nodes share a set of properties. In the context of lesion-deficit mapping, the properties of interest are the presence of damage, the associated cognitive deficit and nuisance factors that could confound their relations. Bayesian stochastic block modelling^10^ is used to infer the hierarchical organization of subnetworks of voxels exhibiting dependence on the behavioural score, disentangled from the incidental spatial structure of lesions. Stochastic block models are non-parametric probabilistic statistical models of the network structure of graphs that enable robust inference to distinct patterns of connectivity arising as a hierarchy of ‘blocks’ or ‘communities’ within them.^48,49^ In the context of a graph model of the lesioned brain, such communities may be shaped by the neural substrate of the behaviour under study, and/or the anatomical patterns of damage. Layered stochastic block models allow us to disentangle these two distinct types of node connectivity, allowing us to isolate the neural dependents of the task from the incidental pathological structure of the lesions. The procedure is described and validated elsewhere and summarized in the Supplementary material.

## Results

### Demographics and background tests

The Frontal, Posterior and HC groups were well-matched for age, sex and years of education (all *P* > 0.05; see Supplementary Table 1). The proportion of tumour or stroke patients did not differ significantly between Frontal and Posterior groups or Left and Right frontal groups (both *P* > 0.05). Similarly, lesion volume did not differ significantly between Frontal and Posterior groups or Left and Right frontal groups (both *P* > 0.05). Lesion volume was not significantly correlated with ART, DRT or RAPM performance, nor was it correlated with performance on different types of ART and DRT questions (all *P* > 0.05).

There were no significant differences between any groups in terms of NART, Incomplete Letters, GNT or TROG scores (all *P* > 0.05; see Supplementary Table 2). The Frontal group had significantly lower scores than the Posterior and HC groups on RAPM [*F*(2,184) = 20.11; *P* < 0.001], S fluency [*F*(2,151) = 19.79; *P* < 0.001] and Stroop [*F*(2,150) = 10.80; *P* < 0.001] tests.

A right frontal effect was observed for RAPM performance: the Right frontal group was significantly more impaired than the Left frontal group (*P* < 0.05). Overall, 181 of 247 patients completed the RAPM. Of these, 116 were included in our previous RAPM article^10^ and 65 were newly recruited. This sample of 65 patients included 14 with focal right frontal and 10 with left frontal lesions. Reassuringly, in these patients, those with right frontal lesions (mean = 5.64 ± 2.10 SD) had lower RAPM scores than patients with left frontal lesions (mean = 7.30 ± 2.50 SD).

Consistent with previous findings,^50,51^ the Left frontal group was significantly more impaired than the Right frontal group on the S fluency and Stroop tests (*P* < 0.05 and *P* < 0.01, respectively).

### Analogical reasoning task

On the Perceptual Baseline Matching Task, there were no significant differences between the Frontal, Posterior and HC groups (see Table 1). In contrast, for overall ART performance, there was a significant difference [*F*(2,238) = 18.93; *P* < 0.001], where the Frontal group was significantly impaired relative to the Posterior and HC groups (both *P* < 0.001). An analysis of the lateralized effects showed that the Right frontal group was significantly more impaired than the Left frontal group (*P* < 0.05).

**Table 1:** Performance on the Analogical Reasoning Task and the Perceptual Matching Baseline Task

	HC (*n* = 72)	Frontal (*n* = 100)	Posterior (*n* = 70)	Left frontal (*n* = 46)	Right frontal (*n* = 54)	Left posterior (*n* = 31)	Right posterior (*n* = 39)
Perceptual Matching Baseline Task (% correct), mean (SD)	98.61 (5.65)	96.62 (7.19)	97.50 (5.34)	97.15 (7.23)	96.17 (7.20)	98.40 (3.25)	96.79 (6.50)
Analogical Reasoning Task (% correct), mean (SD)	79.40 (11.93)	67.79^a,^***^,b,^*** (15.51)	77.38 (13.51)	73.19 (16.05)	63.19^c,^** (13.56)	78.63 (11.87)	76.39 (14.76)
‘Progression’ rule questions (% correct), mean (SD)	83.22 (12.86)	71.94 ^a,^***^,b,^*** (16.60)	81.19 (14.52)	75.22 (16.68)	69.14 (16.16)	81.99 (14.54)	80.55 (14.73)
‘Odd-one-out’ rule questions (% correct), mean (SD)	75.58 (15.09)	63.71^a,^** ^,b,^*** (20.06)	73.57 (17.08)	71.29 (19.29)	57.25^c,^** (18.53)	75.27 (14.19)	72.22 (19.15)

Groups are compared using analysis of covariance (ANCOVA), controlling for age. Scores with significant *P*-values are in bold and **P < 0.01; ***P < 0.001. For comparison between Frontal, Posterior and Healthy control (HC) groups, these reflect corrected *P*-values.

SD = standard deviation.

^a^Significant difference from Posterior group.

^b^Significant difference from HC group.

^c^Significant difference from Left frontal group.

For the Progression rule, there was a non-lateralized frontal effect. Thus, the Frontal group was significantly impaired relative to the Posterior and HC groups [ANCOVA: *F*(2,238) = 15.20; *P* < 0.001; *post hoc* tests: both *P* < 0.001]. The performances of the Left and Right frontal groups did not differ significantly.

For the OOU rule, there was a right frontal effect. Thus, the Frontal group was significantly impaired relative to the Posterior and HC groups [ANCOVA: *F*(2,238) = 11.74; *P* < 0.001; *post hoc* tests: *P* < 0.01 and *P* < 0.001, respectively; see Table 1]. Importantly, the Right frontal group was significantly impaired relative to the Left frontal group (*P* < 0.01).

To investigate whether the right frontal effect for the overall ART performance was modulated by difficulty, performance was analysed on intra- and cross-dimensional questions separately. Pairwise comparisons confirmed that performance was significantly more accurate on intra-dimensional than cross-dimensional questions in the entire sample (*P* < 0.001) and in Frontal (*P* < 0.05), Posterior (*P* < 0.05) and HC groups separately (*P* < 0.05). Notably, right frontal effects were observed on both intra- and cross-dimensional questions. Thus, on both, the Frontal group was significantly impaired [ANCOVA: *F*(2,238) = 13.21; *P* < 0.001; ANCOVA: *F*(2,238) = 12.45; *P* < 0.001, respectively] relative to the Posterior (both *P* < 0.01) and HC groups (both *P* < 0.001). Importantly, the Right frontal group was impaired relative to the Left frontal group on both intra- and cross-dimensional questions (*P* < 0.05).

We also analysed performance on questions where rules applied to numerical value, colour or size. For both colour and size, the Frontal group was significantly impaired relative to the Posterior [ANCOVA: *F*(2,238) = 6.57; *P* < 0.01; *post hoc* tests: *P* < 0.01 and *P* < 0.05, respectively] and HC groups [ANCOVA: *F*(2,238) = 9.27; *P* < 0.001; *post hoc* tests: *P* < 0.05 and *P* < 0.001, respectively]. For both colour and size, the Right frontal group was significantly impaired relative to the Left frontal group (both *P* < 0.01). For questions where rules applied to the stimuli’s numerical value, there was a non-lateralized frontal effect: the Frontal group was significantly impaired relative to the HCs [ANCOVA: *F*(2,238) = 7.29; *P* < 0.001; *post hoc* test: *P* < 0.001], but the performances of the Right and Left frontal groups did not differ significantly.

### Deductive reasoning task

There was a significant difference between the Frontal, Posterior and HC groups for overall DRT performance [*F*(2,142) = 7.73; *P* < 0.001; see Table 2]. The Frontal group was significantly more impaired relative to the HCs only (*P* < 0.01). The Right and Left frontal groups did not differ significantly.

**Table 2:** Performance on the Deductive Reasoning Task

	HC (*n* = 48)	Frontal (*n* = 55)	Posterior (*n* = 43)	Left frontal (*n* = 24)	Right frontal (*n* = 31)	Left posterior (*n* = 19)	Right posterior (*n* = 24)
Deductive Reasoning Task (% correct), mean (SD)	82.79 (15.22)	71.67^b,^** (15.90)	79.37 (16.03)	75.88 (14.65)	68.42 (16.29)	77.05 (17.83)	81.21 (14.57)
Indeterminate questions (% correct), mean (SD)	76.06 (25.21)	55.89^a,^*^,b,^*** (30.53)	70.56 (27.60)	65.58 (28.25)	48.39^c,^* (30.53)	69.26 (26.18)	71.58 (29.18)
Determinate questions (% correct), mean (SD)	84.79 (15.42)	76.69^b,^* (15.12)	82.14 (15.17)	79.00 (14.65)	74.90 (15.47)	79.53 (17.26)	84.21 (13.30)

Groups are compared using analysis of covariance (ANCOVA), controlling for age. Scores with significant *P*-values are in bold and *P < 0.05; **P < 0.01; ***P < 0.001 (for comparisons between Frontal, Posterior and Healthy control (HC) groups, these reflect corrected *P*-values).

SD = standard deviation.

^a^Significant difference from Posterior group.

^b^Significant difference from HC group.

^c^Significant difference from Left frontal group.

A right frontal effect was also found for indeterminate questions. Thus, the Frontal group was significantly impaired [ANCOVA: *F*(2,142) = 7.93; *P* < 0.001] relative to the Posterior and HC groups (*P* < 0.05 and *P* < 0.001, respectively). Interestingly, the Right frontal group was significantly impaired relative to the Left frontal group (*P* < 0.05). For determinate questions, the Frontal group was significantly impaired relative to the HC group [ANCOVA: *F*(2,142) = 4.23, *P* < 0.05; *post hoc* test: *P* < 0.05] but the Right and Left frontal groups did not differ significantly.

### Neuroimaging analysis

**Figure 2:**
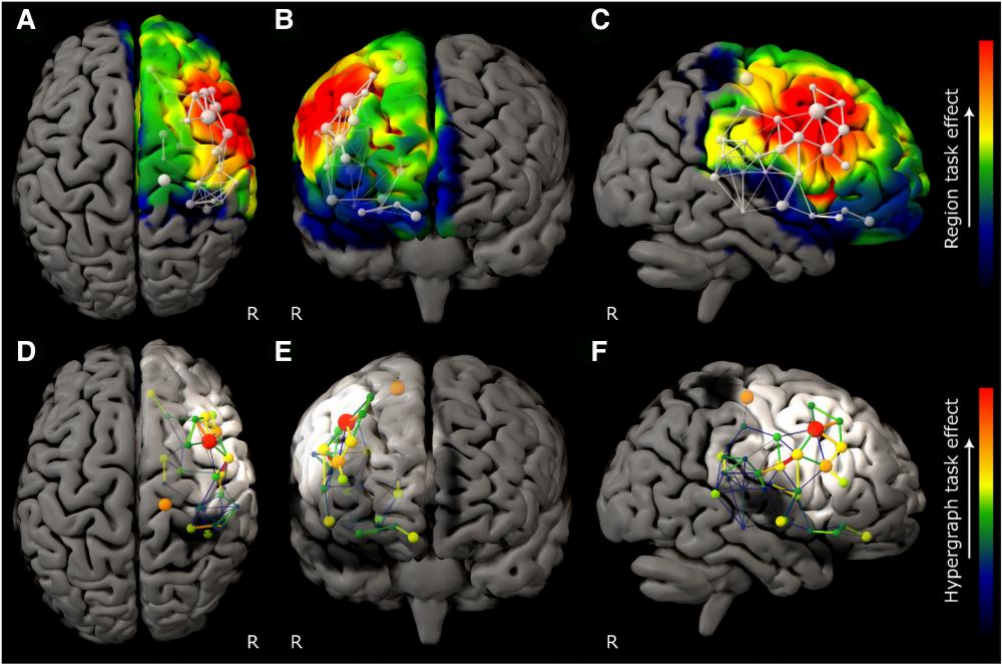
**Graph lesion-deficit mapping of Analogical Reasoning Task performance**. (**A**–**C**) Backprojection of Analogical Reasoning Task (ART) edge weights onto the brain. (**D**–**F**) The hypergraph at the first agglomerative level shows the inter-community relations between brain regions modulated by performance. The colour map for all plots indexes ART edge weight. The test model exhibited lower entropy—515 399 versus 776 413 nats—than the null model, providing inferential support for distinguishing ART from lesion co-occurrence effects (odds ratio of e261 013 in favour of test). Only regions where 95% confidence intervals did not cross those of the lesion weight distribution are shown.

**Figure 3:**
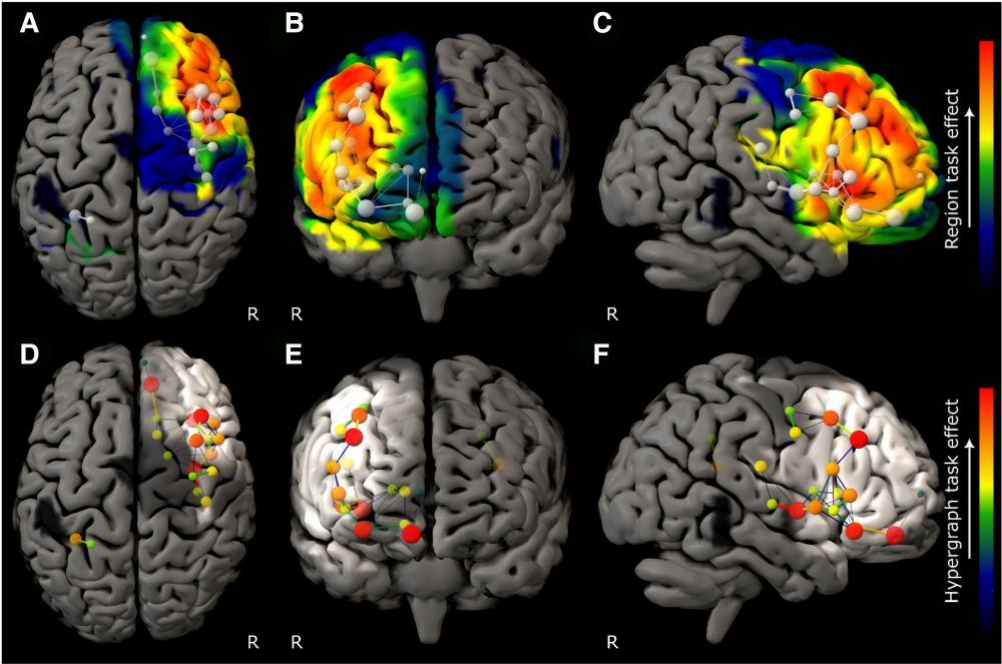
**Graph lesion-deficit mapping of Deductive Reasoning Task performance**. (**A**–**C**) Backprojection of Deductive Reasoning Task (DRT) edge weights onto the brain. (**D**–**F**) The hypergraph at the first agglomerative level shows the inter-community relations between brain regions modulated by performance. The colourmap for all plots indexes DRT edge weight. The test model exhibited lower entropy—476 362 versus 725 748 nats—than the null model, providing inferential support for distinguishing DRT from lesion co-occurrence effects (odds ratio of e249 386 in favour of test). Only regions where 95% confidence intervals did not cross those of the lesion weight distribution are shown.

**Figure 4:**
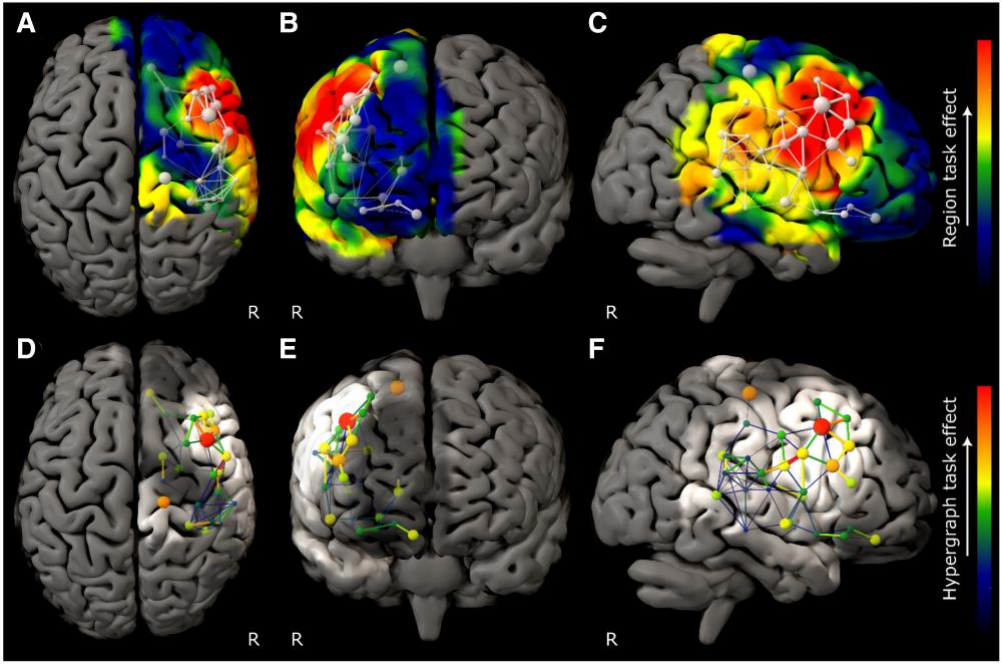
**Graph lesion-deficit mapping of Raven’s Advanced Progressive Matrices performance**. (**A**–**C**) Backprojection of Raven’s Advanced Progressive Matrices (RAPM) edge weights onto the brain. (**D**–**F**) The hypergraph at the first agglomerative level shows the inter-community relations between brain regions modulated by performance. The colourmap for all plots indexes the RAPM edge weight. The test model exhibited lower entropy—451 868 versus 699 072 nats—than the null model, providing inferential support for distinguishing RAPM from lesion co-occurrence effects (odds ratio of e247 204 in favour of test). Only regions where 95% confidence intervals did not cross those of the lesion weight distribution are shown.

The model of ART performance revealed a set of brain communities concentrated in the right middle and inferior frontal gyri, with a hierarchy dominated by caudal middle frontal gyrus (Fig. 2). DRT performance was also strongly right frontal but engaged a more rostral and broadly distributed network spanning inferior and superior frontal gyri (Fig. 3). RAPM revealed a still more extensive right frontal network, dominated by superior frontal gyrus (Fig. 4). The only comparatively remote region, observed in the ART and RAPM networks, was right dorsal post-central gyrus.

**Figure 5:**
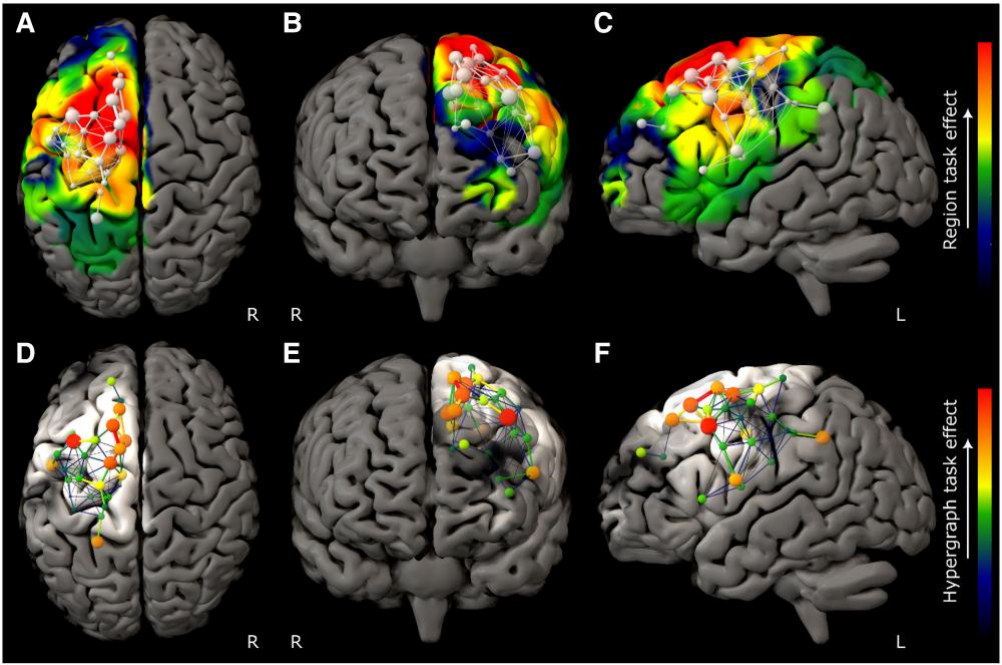
**Graph lesion-deficit mapping of S fluency**. (**A**–**C**) Backprojection of fluency edge weights onto the brain. (**D**–**F**) The hypergraph at the first agglomerative level shows the inter-community relations between brain regions modulated by performance. The colourmap for all plots indexes the S fluency edge weight. The test model exhibited lower entropy—432 339 versus 646 761 nats—than the null model, providing inferential support for distinguishing S fluency from lesion co-occurrence effects (odds ratio of e214 421 in favour of test). Only regions where 95% confidence intervals did not cross those of the lesion weight distribution are shown.

**Figure 6:**
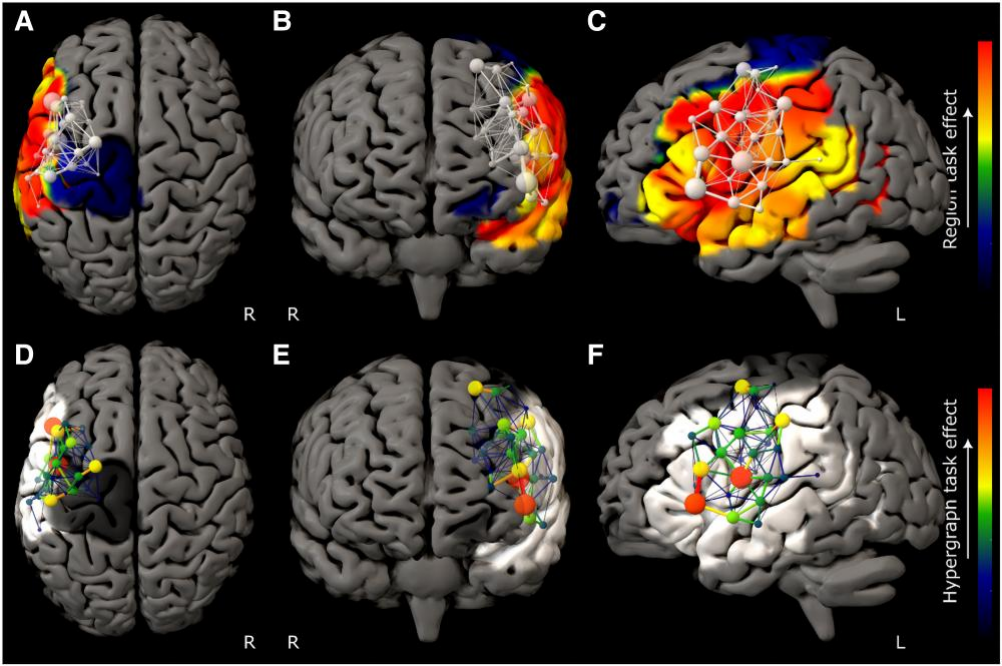
**Graph lesion-deficit mapping of Stroop**. (**A**–**C**) Backprojection of Stroop edge weights onto the brain. (**D**–**F**) The hypergraph at the first agglomerative level shows the inter-community relations between brain regions modulated by performance. The colourmap for all plots indexes the Stroop edge weight. The test model exhibited lower entropy—440 889 versus 671 673 nats—than the null model, providing inferential support for distinguishing Stroop from lesion co-occurrence effects (odds ratio of e230 783 in favour of test). Only regions where 95% confidence intervals did not cross those of the lesion weight distribution are shown.

Modelling S fluency and Stroop in the same cohort yielded strongly left lateralized networks. S fluency engaged left dorsal superior and middle frontal gyri, in keeping with previous studies (see Fig. 5), and Stroop showed an extensive left frontal network centred on the inferior frontal gyrus, again in line with expectations (see Fig. 6).

## Discussion

To our knowledge, this is the first study to investigate the neural substrates of AR and DR in a large cohort of prospectively recruited patients with unilateral focal frontal or posterior lesions and HCs. We analysed performance on two new tests: the ART and DRT and employed graph lesion-deficit mapping.

The results of our behavioural and neuroimaging analyses converged to implicate a right frontal network in ART and DRT performance. On the ART, Right frontal patients were significantly more impaired than Left. On the DRT, Right frontal patients were significantly more impaired than Left on questions with indeterminate conclusions but not determinate ones. Graph lesion-deficit mapping implicated a set of predominantly right frontal brain communities in performance on both tests.

The ART and DRT are clearly complex tasks and, hence, likely to rely upon diverse cognitive abilities. Yet, our finding of shared neural dependence suggested reliance upon a common executive process. While the nature of this process is not immediately obvious, consideration of previous theoretical proposals may shed some light. Prado *et al*.,^24^ on the basis of data from neuroimaging studies in healthy participants, argued that right frontal and bilateral posterior parietal cortices may support performance on relational DR questions. Notably, both our ART and DRT included relational questions. Hence, the our focal lesion study provided a critical test of the necessity of these brain areas for relational reasoning. Interestingly, we found that posterior parietal cortex made no significant contribution to performance on either test, but the right frontal lobe was critical for both.

Prado *et al*.^24^ also suggested that, due to the right hemisphere’s well-known dominance in visuospatial processing, predominantly right lateralized areas may be crucial for relational DR performance. This, they argued, is because relational questions can easily be mapped onto a linear continuum and may, therefore, rely on mental representations that are strongly visuospatial. Hence, one may expect right lateralized effects to be evident on DRT determinate questions, where information can be arranged to form a single linear sequence (e.g. IF Lucy is braver than Samantha, AND Samantha is braver than Rebecca, THEN Lucy is braver than Rebecca?). However, if one follows the logic put forward by Prado *et al*.,^24^ one may not expect right lateralized effects on indeterminate questions because, for these questions, information contained within the premises cannot be mapped onto a linear continuum. For example, for questions such as ‘IF Chris gets better grades than Helen: AND Chris gets better grades than Dorothy; THEN Helen gets better grades than Dorothy?’, the relationships between ‘Chris and Helen and Chris and Dorothy’ are independent and must, therefore, be considered separately. However, contrary to the aforementioned suggestion about the DRT,^24^ we did not find that right frontal patients were significantly impaired relative to left frontal patients for determinate questions. Instead, right frontal patients were significantly impaired relative to those with left frontal lesions for indeterminate questions. Hence, our results broadly agree with the suggestion that the right frontal lobe is critical for relational reasoning questions^24^ but do not support the suggestion that they rely on mental representations requiring visuospatial processing.

An alternative suggestion was put forward by Goel *et al*.,^4^ who suggested that the left and right frontal lobes may be critical to performance on determinate and indeterminate DR questions, respectively. This proposal was based on a small number of patients (*n* < 20), most with traumatic brain injury: typically associated with diffuse axonal injury.^40^ In our large sample of patients with focal unilateral lesion caused by brain tumour or stroke, we found a significant frontal effect but no significant difference between the left and right frontal patients’ performances on determinate questions. Our findings suggest that both left and right frontal regions may support cognitive processes involved in generating deductive conclusions on determinate problems. Future research should investigate this further to explore potential more subtle lateralization effects. Our findings are in broad agreement with those reported by Goel *et al*.,^4^ who found that their nine right frontal patients were impaired in solving DR indeterminate questions. They suggested that the right frontal lobe supports cognitive processes necessary to represent and maintain uncertainty. Notably, an impairment in representing and maintaining uncertainty is unlikely to explain our right frontal patients’ ART performances, since all questions had a determinate solution. Thus, it is plausible that impairment in a different type of executive process from that suggested by Goel *et al*.^4^ may underpin our right frontal patients’ DRT and ART performances. An analysis of performance on different types of ART questions may provide insights into the nature of this process.

On the ART, we found that right frontal patients were more impaired than left on questions that followed the OOU but not Progression rule. Notably, the Progression rule—numbers, colours and size increase/decrease progressively—draws upon sequential numerical or perceptual patterns that participants may have encountered previously. In contrast, the OOU rule—stimuli differ in numerical value, colour or size—is likely to be somewhat novel, necessitating active maintenance of the task rule, which must be applied to the source and target sets in parallel. We suggest that both ART OOU questions and DRT indeterminate questions rely upon the ability to actively maintain several novel representations in parallel, which Shallice and Cipolotti^9^ held to be a process involving the right prefrontal cortex.

Our findings are relevant to influential theories proposing close links between reasoning abilities and Gf. For example, the influential Cattell–Horn–Carroll theory suggests that Gf is a ‘broad’ cognitive domain comprising several qualitatively different reasoning abilities, including AR and DR.^6^ Interestingly, the results of our graph lesion-deficit mapping showed that the right frontal brain areas critical to ART and DRT performance were remarkably similar to the areas critical for RAPM performance, one of the best-established measures of Gf. This finding supports the notion of a close association between Gf, AR and DR abilities. Strikingly, contrary to the P-FIT and MD network theories that have proposed involvement of posterior brain areas in reasoning,^7,8^ we found that ART and DRT performance was unimpaired in posterior patients. Moreover, graph lesion-deficit mapping indicated that the contribution of posterior brain areas was minimal.

Of course, reasoning is complex, and our findings do not imply that other brain areas are not critical for performance on other reasoning tasks. It has been proposed that left frontal areas may be crucial on tests including categorical or propositional DR questions.^24^ Notably, this suggestion was based on neuroimaging evidence, which cannot establish the necessity of brain area for a cognitive function—a limitation that cannot be addressed by further methodological refinement.^29^ While a handful of focal lesion studies on reasoning exist, with the potential to offer greater causal power, these have been limited by small samples,^4,35,36,39^ inclusion of patients with non-focal lesions^4^ and/or the use of VLSM: a methodology with now well-understood flaws.^35-38^ We suggest that our strategy of combining graph-based lesion-deficit mapping with detailed investigation of reasoning abilities in a large sample of focally lesioned patients offers a promising methodological approach that can be applied to investigate performance on other tests of reasoning.

It is important to recognize that topological inference in the human brain—from both lesion and correlative data—is inherently challenging. Functional reorganization may have an impact on neural dependence, both in rapidly evolving lesions evaluated in the chronic phase—the commonest experimental scenario—and less rapidly evolving lesions such as tumours. Though adaptation in the former case may be predicted to be less incremental, it is a limitation common to lesion-deficit studies outside the—usually unfeasible—acute context. Where the causal pathology evolves over time, as with tumours, non-stationary effects may be amplified. Nonetheless, there are to our knowledge no known substantive lateralization or rostrocaudal axis differences in tumour progression that could explain the topological differences here. Moreover, our behavioural findings do not suggest that such differences were present. For example, we report robust right lateralized effects for ART, DRT and RAPM performance and, within the same cohort, strongly left lateralized effects for Stroop and phonemic fluency performance.

For the same reason, though the detectability of a lesion-deficit relation will inevitably depend on the interaction between the lesion distribution—not just anatomical location but the high-dimensional pattern of lesion features—and the distributed structure of the underlying neural substrate, that two independent tasks—Stroop and phonemic fluency—evaluated in the same cohort show robust left lateralization,^50,52-54^ demonstrates that the lesion distribution is adequate for identifying left-sided effects.

Equally, since heterogeneous pathological processes do not necessarily respect lobar or any other crude anatomical boundary, we cannot expect the approximate subdivisions into left, right, frontal and posterior groups—defined by >70% of the lesion falling within the defined territory—to coincide perfectly with the graph lesion-deficit maps. We employ high-resolution, multivariate, brain-wide topological inference here precisely because no *a priori* assumptions can be made about the structure of neural dependents—at any scale—and because the task requires the disentanglement of complex behaviourally relevant and incidental anatomical effects. The lesion-deficit model gives us the best inference to the underlying anatomy.

It is unlikely that our right frontal effects on the ART, DRT and RAPM can be explained by potential lateralized specialization for verbal versus non-verbal material. This is because a right frontal network was implicated in performance not only on the ART but, crucially, also on our entirely verbal, DRT. In our sample, of the 239 patients for whom handedness was recorded, only 2.7% of patients were left-handed. However, given the high prevalence of left hemispheric dominance in left-handers, we could not reliably use handedness as an index of dominance, and this is a limitation of our study. A firm answer to this question regarding functional cerebral asymmetries for language and reasoning will require dedicated investigation of a right language dominant cohort.

It is also implausible that our right frontal effects could have arisen due to general task ‘difficulty’. Consistent with previous findings, we found that Frontal, Posterior and HC groups were more accurate on ART intra- than cross-dimensional trials.^36^ Yet, right frontal patients were significantly less accurate than left on both intra- and cross-dimensional trials.

Our findings suggest that the ART and DRT may provide useful information in clinical practice. To date, only a very small number of clinical tests have been shown capable of detecting right frontal lobe dysfunction.^10,55^ Translating the ART and DRT into clinical practice may address this unmet need. Notably, both tests also lack ceiling effects: an issue that applies to many existing neuropsychological tests.^56^

In conclusion, our study represents the most robust investigation of reasoning abilities in patients with single, focal, unilateral lesions. Combining detailed analysis of performance on novel reasoning tests with graph-based lesion-deficit mapping in a large cohort has provided novel insights into the neural basis of reasoning. Our findings imply that a right frontal network is critical for aspects of AR and DR. They also suggest that the ART and DRT are promising new tests, capable of evaluating reasoning abilities and identifying right frontal lobe dysfunction.

## Supplementary Material

awaf062_Supplementary_Data

## Data Availability

The data that support this study are available from L.C., upon reasonable request. The code for replicating the analysis will be made openly available at https://github.com/high-dimensional/reasoning.
