# Supplementary material for: A right frontal network for analogical and deductive reasoning
Source: Brain. 2025 Apr 16;148(5):1757–68. doi: 10.1093/brain/awaf062 (PMC12073978; doi:10.1093/brain/awaf062)
Supplement: awaf062_Supplementary_Data [file awaf062_supplementary_data.pdf]

Supplementary Materials

Participants

Patients were recruited from inpatient and outpatient stroke and neuro-oncology services at the National Hospital for Neurology and Neurosurgery between the 15th of March 2018 and 29th of September 2022. Inclusion criteria were: i) no history of psychiatric disorders, alcohol or substance abuse, or other neurological disorders; ii) ability to speak and understand English; iii) presence of a focal stroke or tumour; iv) age between 18– 80 years; and v) absence of gross perceptual (no neglect, >5th cut-off on the Incomplete Letters test; Warrington, 1991) or language impairments (>5th %ile on the Graded Naming Test, GNT; McKenna & Warrington, 1980). Age at assessment, gender, and years of education were recoded.

Neuroimaging investigations

MRI and CT scans were obtained during routine clinical care. MRI scans were obtained on either a 3 T or 1.5 T Siemen scanners following a diversity of clinically- determined protocols outside our control. CT studies were obtained on Toshiba or Siemens spiral scanners. Note that since the input to the lesion-deficit models is not raw image data but comparatively large, manually-traced, binary lesion masks, we assumed—as is standard in the field—that the effect of variations in acquisition parameters is likely negligible and need not be explicitly modelled. Lesions were traced and independently classified using MIPAV (https://mipav.cit.nih.gov/) by J.M., E.C. and checked by P.N., who was blind to the study results. Registration of every lesion in template space was checked by J.M. or E.C. and confirmed by P.N. and J.R The lesion masks were non-linearly normalized to Montreal Neurological Institute (MNI) stereotaxic space at 2×2×2 mm resolution using SPM-12 software with enantiomorphic correction (http://www.fil.ion.ucl.ac.uk; see Nachev et al., 2008).

Analogical Reasoning Task and Perceptual matching baseline task

The Perceptual matching baseline task was administered first. This began with three instruction questions, where the correct answers were demonstrated (see Supplementary Figure 1a). On the first instruction question participants were shown a set of three coloured numbers on the left of the screen (the source set) and two sets of three coloured numbers on the right of the screen (the target sets). Participants were told: "*Here you can see one set of numbers on the left and two on the right. In the first part of this test, I would like you to touch the set of numbers on the right that looks most similar to the set of number on the left. For example, here you can see that, out of these two sets of numbers on the right, this one (examiner points to the numbers on the bottom right) looks most similar to the set of numbers on the left (examiner point to the numbers on the left). This is because both sets of numbers are in a big font size. Whereas the numbers here (examiner points to the numbers on the top right) are in a small font size. So, this is the correct answer (examiner points to the numbers on the bottom right).*

Participants were then presented with two more instructions questions, where the sets of numbers in the correct target set matched the numbers in the source set in terms of numerical values and colour (see Supplementary Figure 1a). Following the instruction questions, participants were administered 12 test questions. Correct answers were based on stimuli's numerical value (n = 4), colour (n = 4) and size (n = 4).

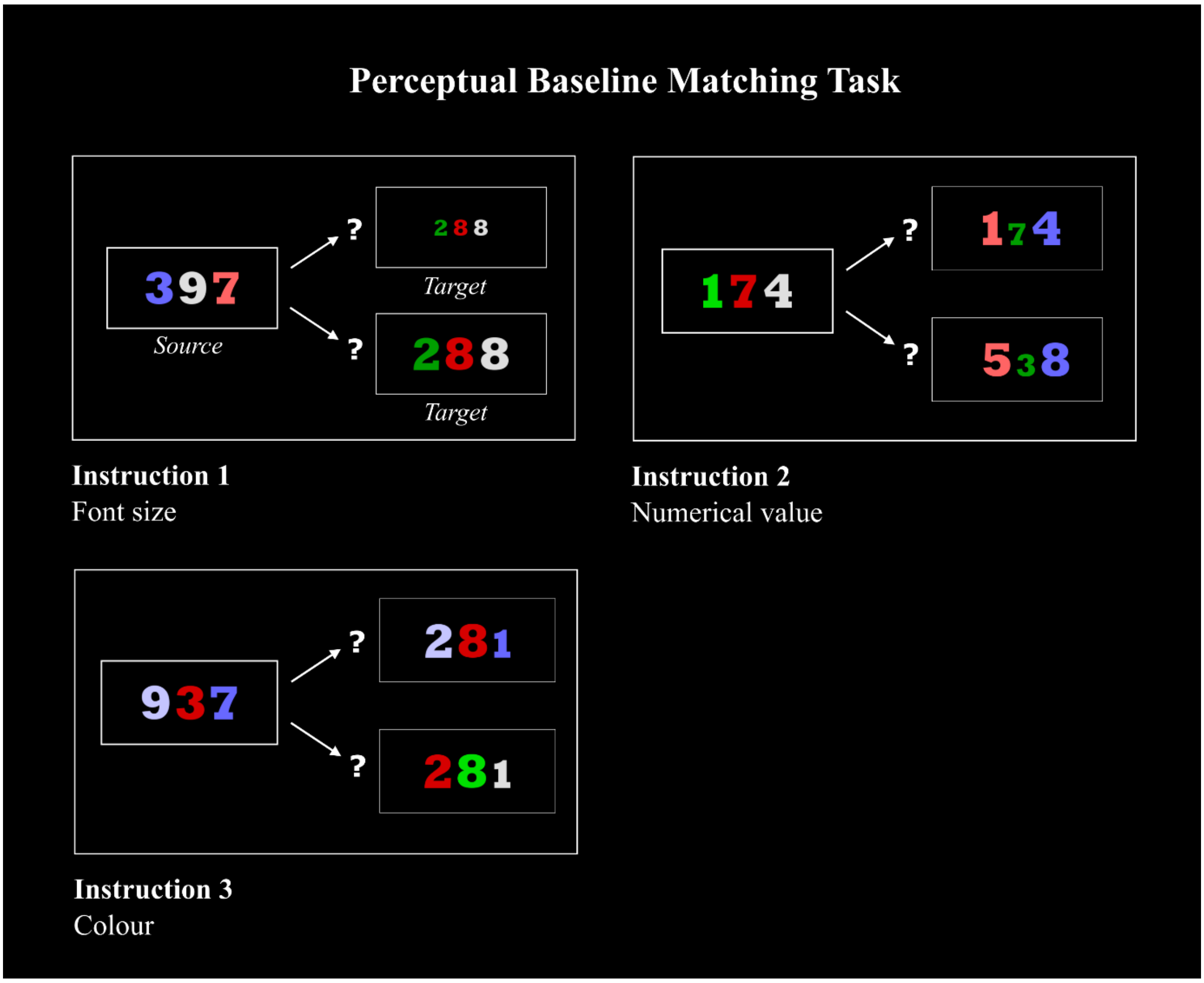

Supplementary Figure 1a. Instruction questions on the Perceptual matching baseline task.

The ART was administered immediately after the Perceptual matching baseline task. To introduce the ART, participants were told: "*This is the second part of the test. Now I would like you to do something different. As before, I would like you to say which of the sets of numbers on the right is most similar to the set of numbers on the left. However, this time you should do this based on whether they follow the same abstract rule, rather than whether they look similar".* Hence, the key difference between the tasks was that on the ART participants were instructed to select a response by using two abstract rules. These were the 'Progression' rule and 'Odd-one-out' (OOU) rule (illustrated in the examples below).

The ART began with six instruction questions, where the Progression and OOU rules were introduced and the correct answers demonstrated (see Supplementary Figure 1b). The first instruction question introduced the Progression rule. Participants were told: "*Here you can see that the numbers on the left are increasing, so there's a progression (examiner points to numbers '1,2,3' on the left). And here you can see that the numbers on the top right of the screen are also increasing, so there is also a progression (examiner points to the numbers '7,8,9' in the top right). But the numbers in the bottom right of the screen are not increasing, so there is no progression (examiner points to the numbers '8,4,7' on the bottom right). So, this is the correct answer, because the numbers are progressing (examiner touches the top right).*

The second instruction question introduced the OOU rule. Participants were told: "*Here you can see a set of numbers on the left. The first number is a different size from the other two numbers (examiner points to the numbers on the left, where the first number is presented in a larger font size than the others). And here you can see that in one of these sets of numbers (examiner points to the sets of numbers on the right), the first number is also a different size from the others, so it's also the odd one out (examiner points to the set of numbers on the bottom right). So, this is the correct answer (examiner points the bottom right).*" The third and fourth instruction questions demonstrated the OOU and Progression rules again (see Supplementary Figure 1b). On both questions the rules applied to the colour of the numbers.

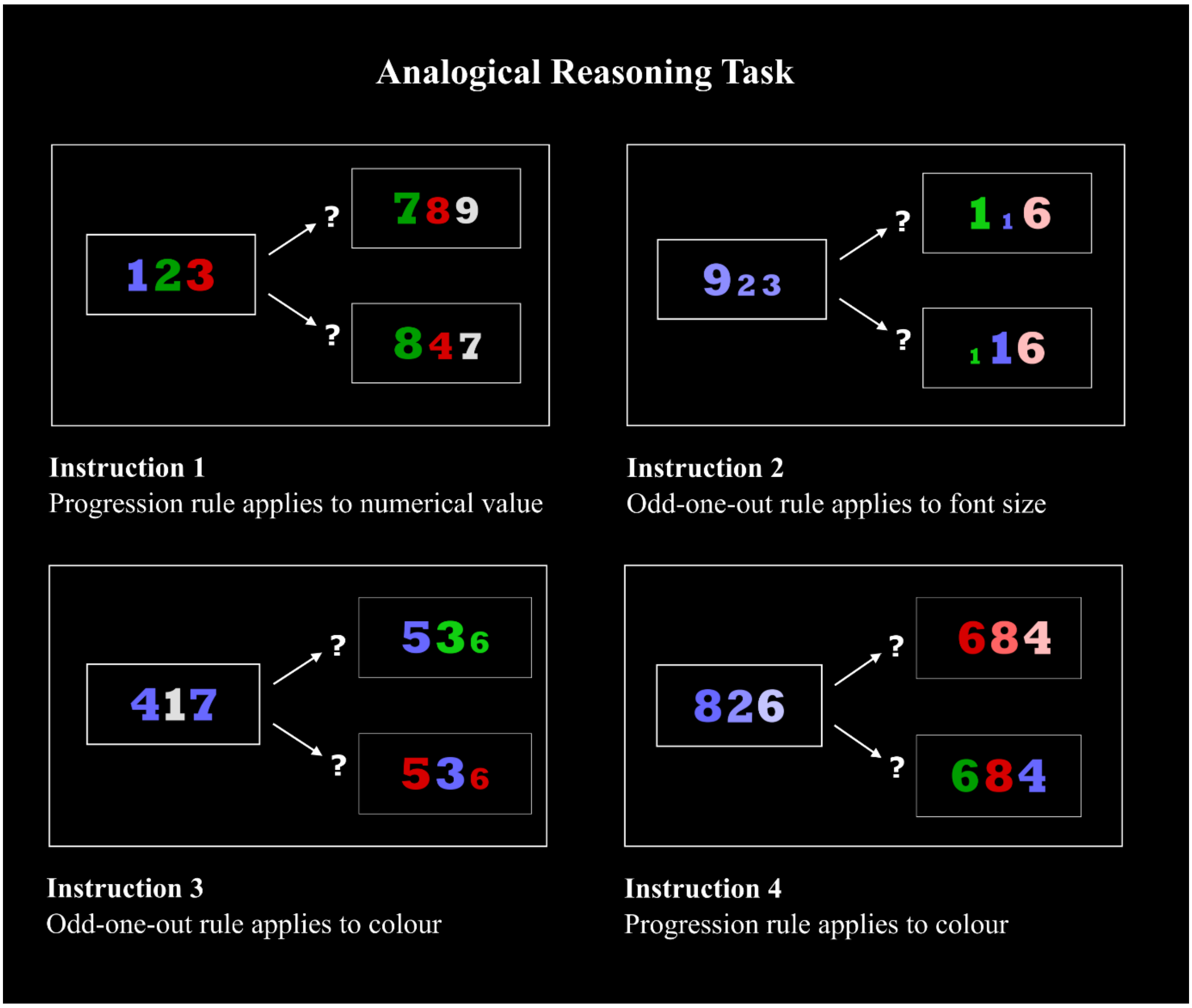

Supplementary Figure 1b. Instruction questions one to four on the ART

Two further instruction questions were then administered to demonstrate examples of 'cross-dimensional' questions i.e. questions when the rule applies to different stimuli features in the source and target sets (e.g. the rule applies to font size in the source set and to colour in the correct target set; see Supplementary Figure 1c; Urbanski et al., 2016). This contrasts with the first four instruction questions, which were 'intra-dimensional' i.e. questions when the rule applies to the same stimuli feature in both the source and correct target set (e.g. the rule applies to colour in both sets). At the start of the fifth instruction question, participants were told: *"Now, these kinds of questions are a bit different. Here you can see that the middle number is*

the odd one out (examiner points to the left), because it's a different <u>*size*</u> *from the other two. And here (examiner points to the bottom right) you can see that the middle is also the odd one out, because the middle number is a different* <u>*colour*</u> *from the other two numbers. So, both here (examiner points to the left) and here (examiner points to the bottom right), the middle number is the odd one out. Whereas here (examiner points to the top right), the middle one is not the odd one out. So, this is the correct answer (examiner points to the bottom right)."* The sixth instruction question was then administered to demonstrate a cross-dimensional question using the Progression rule (see Supplementary Figure 1c.).

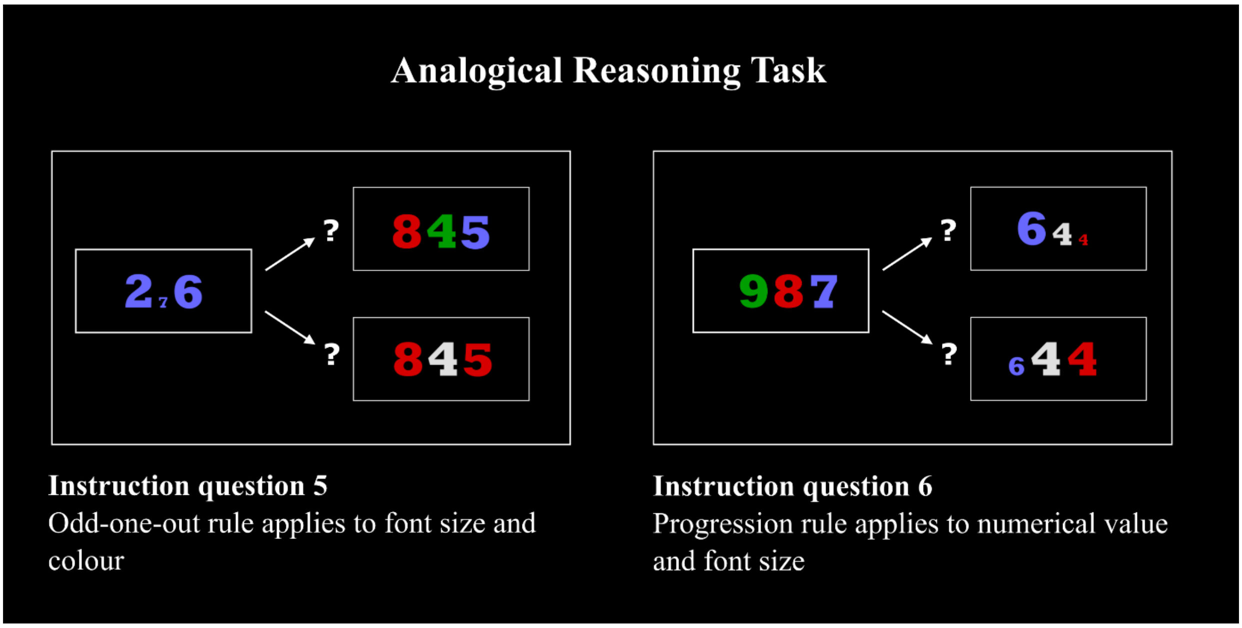

Supplementary Figure 1c. Instruction questions five and six on the ART.

Following the six instruction questions, participants were then administered 24 test questions. They were instructed to respond by touching whichever target set followed the same abstract rule as the source set, and to respond as quickly and accurately as possible. Half of the questions followed the Progression rule (n = 12) and half followed the OOU rule (n = 12). Both rules applied to the stimuli's numerical value (n = 8), colour (n = 8) and size (n = 8). Half of the questions were intra-dimensional and half were cross-dimensional, which were counterbalanced across the Progression and OOU rules and the stimuli's numerical value,

colour and size. On both tasks, stimuli appeared on-screen for one minute or until participants touched a target set. Following a response, a black screen appeared for 500 milliseconds, followed by a red cross or green tick that appeared for one second.

Deductive Reasoning Task

The DRT comprised a series of 24 relational deductive reasoning questions (e.g. *IF Sam is happier than Tom, AND Tom is happier than Jack, THEN Sam is happier than Jack?).* Participants were told: *"On each question, you will be presented with a series of three sentences. Your task is to determine if the third sentence follows logically from the first two sentences. If it does follow logically, press Yes. Otherwise, press No".*

Participants were then presented with two practice questions. The first practice question was logically valid - the third sentence followed logically from the first two sentences - and so the correct answer was 'Yes'. This question had a determinate solution (e.g. *IF Lucy is braver than Samantha, AND Samantha is braver than Rebecca, THEN Lucy is braver than Rebecca?*). The second question was logically invalid - the third sentence did not follow logically from the first two sentences – so the correct answer was 'No'. This had an indeterminate solution (e.g. *IF Chris gets better grades than Helen: AND Chris gets better grades than Dorothy; THEN Helen gets better grades than Dorathy?*). Feedback, in the form of the word "Correct" or "Incorrect" appeared on-screen after both practice questions. If participants made an error on either practice question, both questions were readministered.

Participants were then administered 24 test questions. These questions were presented in the same format as the practice questions, with no feedback provided. Participants were instructed to indicate, as quickly and accurately as possible, whether each question was logically valid by pressing either the words 'Yes' or 'No'. There was an equal number of valid and invalid questions. All valid questions were necessarily determinate. Following the design

of Goel and colleagues (2007), half of the invalid questions were determinate and half were indeterminate. Questions were presented in the centre of the screen in white size 16 Times New Roman font, against a black background. The words ‘Yes’ and ‘No’ appeared immediately underneath each sentence and all stimuli remained on-screen for one minute or until a response was selected.

Graph lesion-deficit mapping

The graph of pairwise relations between anatomical loci at high resolution is too large to be computationally tractable. The imaging data was therefore resampled to 4x4x4 mm resolution, extracting an adjacency matrix for each patient. We deliberately did not use a standard *a priori* parcellation to avoid potential bias from the parcellation itself, as well as to infer a higher resolution map than any current parcellation permits (see Cipolotti et al., 2023; Supplementary Figure 1). We then constructed an undirected, weighted graph combining all individual lesion graphs across all patients. This network comprised nodes corresponding to all voxels of the brain, with edges between all co-lesioned voxels. Iterating in separate models across, ART, DRT, RAPM, S fluency, and Stroop, edges were weighted by two variables: the count of the number of times a voxel and an adjacent neighbor were damaged together —a lesion co-occurrence weight— and the inverse of the patient’s score in each neuropsychological test. The graph was undirected, as the direction of any relationship between collaterally lesioned areas is not informed by the data at hand.

We filtered edges to limit analysis to the top 50% connected nodes, removing edges with fewer than 3 connections, where sampling was too low to support robust inference. There were no node self-loops. We rescaled both lesion co-occurrence and neuropsychological test edge weights to the range 0 to 1, manually reviewing all edge weight histograms to validate our choice of prior distribution in the Bayesian model.

For each cognitive test, we proceeded to evaluate the hierarchical community structure of the network with a non-parametric Bayesian hierarchical weighted stochastic block model incorporating layered and attributed properties implemented in graph-tool (https://graph-tool.skewed.de). We began by fitting a null model, with the two kinds of edge weight—neuropsychological score and lesion co-occurrence—randomly distributed across two layers. We then fitted a test model with each type of weight assigned to its own specific layer. Neuropsychological weights were modelled as Gaussian; lesion co-occurrence weights as Poisson distributions. Having initialised a fit, we used simulated annealing to further optimise it, with a default inverse temperature of 1 to 10. To ensure that equilibration was driven by changes in the entropy criterion, we did not specify a finite number of draws but used a wait step of 100 iterations for a record-breaking event. Model entropy was used to determine if the layered model fit was better than the null, indicating that the inferred community structure distinguished the neuropsychological variable and lesion co-occurrence effects. The incident edge weights of blocks with greater task vs lesion weighting based on 95% confidence intervals for a pair-wise comparison were backprojected onto the brain, as well as the first agglomerative levels of the community hierarchy. In keeping with other studies in the field (Cipolotti et al., 2023; Gläscher et al., 2009), we explicitly chose not to model aetiology, having previously shown this nonadditive for model performance.

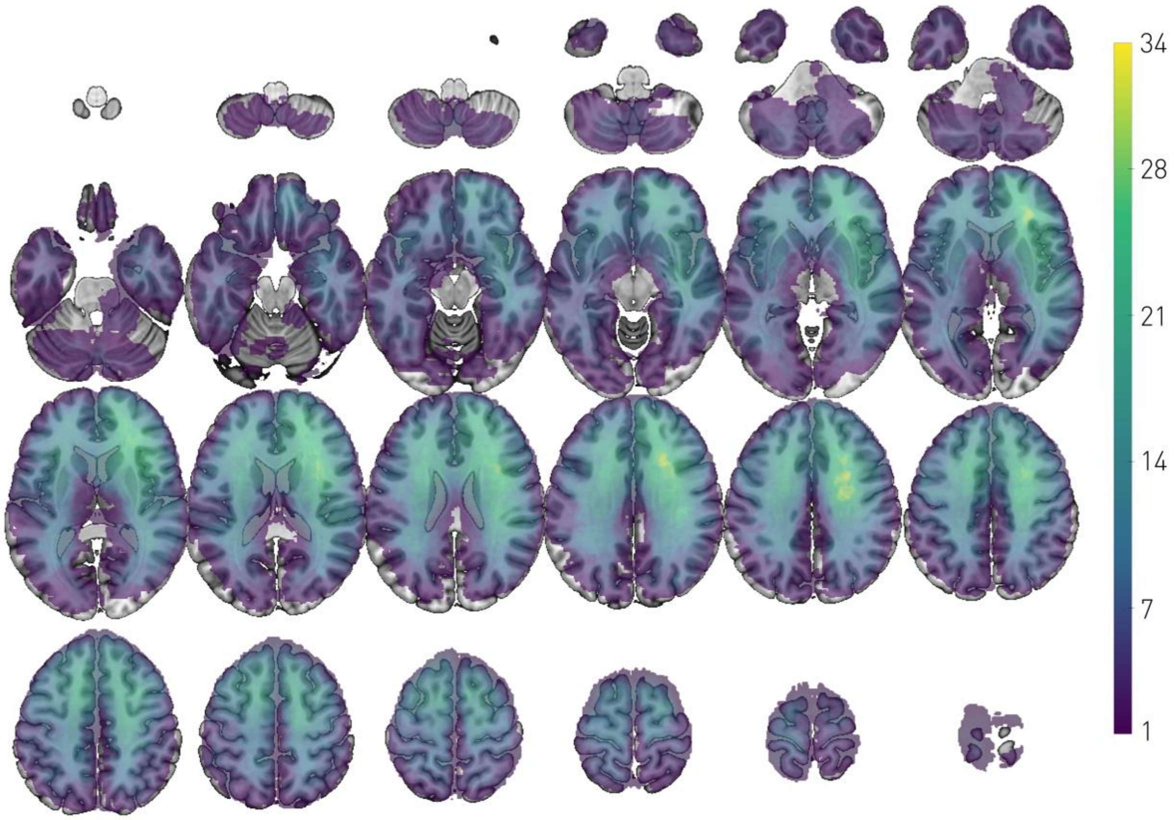

Supplementary Figure 2. Lesion distribution map. Voxel-wise sum of the 237 modelled lesions overlaid on the MNI152 T1 template distributed with MRIcroGL(https://www.nitrc.org/projects/mricrogl). The images are displayed in neurological convention (left is left), and the colourmap ranges from 1 to the image maximum.

Supplementary Table 1. Demographics

	HC<br>(*N* = 81)<br>Mean	Frontal<br>(*N* = 102)<br>Mean	Posterior<br>(*N* = 74)<br>Mean	Left Frontal<br>(*N* = 47)<br>Mean	Right Frontal<br>(*N* = 55)<br>Mean	Left Posterior<br>(*N* = 32)<br>Mean	Right Posterior<br>(*N* = 42)<br>Mean
Age (years)	48.38	47.52	45.99	44.83	49.82	48.31	44.21
(SD)	(15.62)	(15.05)	(15.71)	(15.59)	(14.31)	(14.31)	(14.71)
Gender (male/female)	35/46	56/48	40/34	26/21	30/25	19/13	21/21
Education (years)	15.26	14.72	15.46	14.78	14.67	15.12	15.70
(SD)	(2.54)	(3.84)	(2.88)	(3.49)	(4.16)	(2.88)	(2.90)
Aetiology (Tumour/stroke/abscess/AVM)		78/19/2/3	54/16/2/2	38/7/0/2	40/12/2/1	21/9/1/1	33/7/1/1
Chronicity (days)		337.39	352.81	302.24	364.07	229.41	455.06
(SD)		(830.91)	(646.84)	(828.81)	(839.28)	(390.97)	(790.98)
Lesion volume (cm^3)		57.32	43.36	56.65	57.95	43.57	43.21
(SD)		(79.16)	(37.59)	(101.94)	(50.46)	(40.06)	(36.26)

HC = Healthy Controls; SD = standard deviation

There are no significant differences.

Supplementary Table 2. Performance on neuropsychological assessment.

	HC<br>Mean	Frontal<br>Mean	Posterior<br>Mean	Left Frontal<br>Mean	Right Frontal<br>Mean	Left Posterior<br>Mean	Right Posterior<br>Mean
NART IQ	106.00	106.72	109.25	105.31	107.93	108.95	109.42
(SD)	(9.63)	(10.07)	(9.41)	(10.55)	(9.61)	(9.54)	(9.48)
VOSP IL (/20)	19.61	19.37	19.40	19.51	19.25	19.29	19.48
(SD)	(0.59)	(0.86)	(0.82)	(0.80)	(0.89)	(0.86)	(0.80)
GNT (/30)	20.12	18.90	20.01	17.52	20.06	19.17	21.13
(SD)	(5.19)	(4.35)	(5.59)	(3.92)	(4.41)	(6.06)	(4.54)
TROG (/12)	9.75	9.41	10.00	9.23	9.58	9.88	10.10
(SD)	(1.60)	(1.65)	(1.65)	(1.50)	(1.80)	(1.65)	(1.67)
RAPM (/12)	9.04	**6.76 a** b***	8.13	7.56	**6.14 c***	7.95	8.28
(SD)	(2.10)	**(2.45)**	(2.27)	(2.44)	**(2.31)**	(2.01)	(2.51)
S fluency (no. words)	18.43	**12.51 a*** b***	16.64	**11.13 d***	13.70	15.23	17.28
(SD)	(5.30)	**(5.22)**	(4.68)	**(5.97)**	(4.20)	(3.75)	(4.98)
Stroop (no. correct)	108.67	**87.29 a* b***	102.84	**77.43 d***	94.20	94.00	108.41
(SD)	(20.09)	**(31.12)**	(32.44)	**(33.10)**	(28.04)	(35.67)	(29.57)

Note: groups are compared using ANCOVA, controlling for age. HC = Healthy Controls; SD = standard deviation; GNT = Graded Naming Test; VOSP IL = Visual Object and Space Perception Battery Incomplete Letters; SS = scaled score; TROG = Test for Reception of Grammar (Bishop, 1989); RAPM = Raven's Advanced Progressive Matrices. Scores with significant *p* values are in bold and starred.

* p <.05; ** p <.01; *** p <.001 (For comparison between Frontal, Posterior and Healthy Control groups, these reflect corrected p values).

a indicates significant difference from posteriors.

b indicates significant difference from healthy controls

c indicates significant difference from left frontals.

d indicates significant difference from right frontals.

References

Cipoloti, L., Ruffle, J. K., Mole, J., Xu, T., Hyare, H., Shallice, T., Chan, E., & Nachev, P. (2023). Graph lesion-deficit mapping of fluid intelligence. *Brain*, *146*, 167–181.

Cipolotti, L., Ruffle, J. K., Mole, J., Xu, T., Hyare, H., Shallice, T., Chan, E., & Nachev, P. (2023). Graph lesion-deficit mapping of fluid intelligence. *Brain*, *146*(1), 167-181. https://doi.org/10.1093/brain/awac304

Gläscher, J., Tranel, D., Paul, L. K., Rudrauf, D., Rorden, C., Hornaday, A., Grabowski, T., Damasio, H., & Adolphs, R. (2009). Lesion mapping of cognitive abilities linked to intelligence. *Neuron*, *61*(5), 681-691. https://doi.org/10.1016/j.neuron.2009.01.026

Goel, V., Tierney, M., Sheesley, L., Bartolo, A., Vartanian, O., & Grafman, J. (2007). Hemispheric specialization in human prefrontal cortex for resolving certain and uncertain inferences. *Cereb Cortex*, *17*(10), 2245-2250. https://doi.org/10.1093/cercor/bhl132

McKenna, P., & Warrington, E. (1980). *The Graded Naming Test*. NFER-Nelso.

Nachev, P., Coulthard, E., Jäger, H. R., Kennard, C., & Husain, M. (2008). Enantiomorphic normalization of focally lesioned brains. *NeuroImage*, *39*(3), 1215-1226. https://doi.org/https://doi.org/10.1016/j.neuroimage.2007.10.002

Urbanski, M., Brechemier, M. L., Garcin, B., Bendetowicz, D., Thiebaut de Schotten, M., Foulon, C., Rosso, C., Clarencon, F., Dupont, S., Pradat-Diehl, P., Labeyrie, M. A., Levy, R., & Volle, E. (2016). Reasoning by analogy requires the left frontal pole: lesion-deficit mapping and clinical implications. *Brain*, *139*(Pt 6), 1783-1799.

Warrington, E. K. (1991). *The Visual Object and Space Perception Battery* Thames Vally Test Company
